# Factors Influencing Health Behavior Adherence in Young and Middle-Aged Stroke Patients

**DOI:** 10.31083/RN51282

**Published:** 2026-09-02

**Authors:** Qiaohui Ye, Yanfei Xu

**Affiliations:** ^1^Department of Acupuncture and Rehabilitation, Hangzhou Hospital of Traditional Chinese Medicine, 310002 Hangzhou, Zhejiang, China

**Keywords:** stroke, patient compliance, rehabilitation, cognitive function, pain, logistic models

## Abstract

**Background:**

Health behavior adherence (HBA) is important for effective rehabilitation and secondary prevention after stroke. Young and middle-aged patients may face particular challenges in maintaining recommended health behaviors during hospitalization because of functional impairment, cognitive deficits, pain, and competing social or occupational responsibilities. However, factors associated with HBA in this population remain insufficiently characterized, and practical tools for the early identification of patients at risk of suboptimal adherence are limited. To identify factors associated with HBA in young and middle-aged patients with stroke during hospitalization and to develop a combined predictive model for the early identification of patients with non-high adherence.

**Methods:**

A single-center retrospective observational study was conducted, including 298 young and middle-aged stroke patients hospitalized between January 2021 and June 2024. Patients were categorized into a high-adherence group and a non-high-adherence group based on the HBA Index. Differences in demographic and functional characteristics between the groups were compared. Correlation analysis and multivariate logistic regression were performed to identify independent factors affecting HBA. Receiver operating characteristic (ROC) curves were constructed to evaluate the predictive performance of individual indicators and the combined model.

**Results:**

Among the 298 patients, 143 (48.0%) were classified as non-high-adherence. The high-adherence group had significantly better functional and cognitive scores, including the Barthel Index (BI), Mini-Mental State Examination (MMSE), Fugl-Meyer Assessment (FMA), and Berg Balance Scale (BBS), and experienced less pain (all *p* < 0.001). Adherence was positively correlated with BI, MMSE, FMA, and BBS scores, and negatively correlated with Numeric Rating Scale (NRS) pain scores. In multivariate logistic regression analysis, MMSE, NRS, educational level, and first stroke status were independently associated with non-high adherence. In the final parsimonious multivariable logistic regression model, MMSE score, NRS score, educational level, and first-stroke status were independently associated with non-high adherence. The final four-predictor model demonstrated good discrimination.

**Conclusions:**

A multidimensional combined predictive model demonstrated good discrimination performance and may help to identify patients at higher risk of non-high adherence.

## 1. Introduction

The recent incidence of stroke among young and middle-aged adults has been rising annually, both globally and in China, affecting not only the core working population and primary family providers but also imposing substantial long-term disability and economic burden [1,2,3]. With continuous improvements in acute-phase management, an increasing number of patients survive and enter the rehabilitation stage. However, the quality of functional recovery and long-term prognosis depends not only on disease characteristics and treatment quality [4] but also, to a considerable extent, on patients’ adherence to health behaviors during hospitalization and subsequent rehabilitation.

Health behavior adherence (HBA) is a fundamental component of secondary stroke prevention and rehabilitation management [5]. Inadequate adherence not only diminishes the effectiveness of rehabilitation training but also increases the risk of stroke recurrence and long-term disability [6,7]. Compared with stroke occurring in older adults, acute ischemic stroke in young adults presents distinct clinical characteristics, including different distributions of vascular risk factors, etiological subtypes, stroke severity patterns, and long-term outcomes. Young stroke patients have a higher likelihood of non-atherosclerotic mechanisms, cardioembolic sources, arterial dissections, and cryptogenic causes, while the disease often occurs during periods of active employment and family responsibility [8]. These differences may influence patients’ perceptions of illness, rehabilitation participation, and health-related behavioral decisions. Nevertheless, most existing studies have focused on elderly stroke populations, with relatively limited attention to young and middle-aged patients. This population exhibits psychosocial characteristics distinctly different from those of older adults: they face greater occupational pressures, family responsibilities, and social roles, while also possessing stronger personal motivation and independent decision-making ability. These factors make the formation mechanisms of HBA potentially more complex and heterogeneous [9,10,11]. Previous studies have suggested that disability, cognitive function, and educational level may be associated with medication adherence or rehabilitation participation after stroke. Pain may additionally act as a practical barrier to rehabilitation participation, although direct evidence regarding its association with multidimensional inpatient adherence remains limited [12,13], yet current evidence remains limited, and systematic, quantitative analyses are lacking.

In view of this, the present study employs a retrospective observational design to systematically evaluate the status of HBA among young and middle-aged stroke patients during hospitalization. We analyze the influencing factors, with a particular focus on functional recovery, cognitive ability, pain intensity, and educational level, and further construct a predictive model. The aim is to provide an evidence-based foundation for accurately identifying high-risk patients and developing targeted intervention strategies in clinical practice.

## 2. Materials and Methods

### 2.1 Study Design

This study employed a single-center retrospective observational design. We retrospectively collected clinical data, functional assessments, cognitive evaluations, pain scores, and rehabilitation-related information from young and middle-aged patients with stroke during hospitalization. The study aimed to identify factors associated with HBA and develop an exploratory predictive model for non-high adherence. Patients with incomplete essential clinical information required for HBA assessment or predictive model construction were excluded. After eligibility screening, all included patients had complete data for the variables analyzed in this study. Therefore, complete-case analysis was performed without imputation. This retrospective observational study was reported in accordance with the Strengthening the Reporting of Observational Studies in Epidemiology (STROBE) guidelines (**Supplementary Material**)

### 2.2 Study Population

#### 2.2.1 Inclusion Criteria

(i) For the purposes of this study, young and middle-aged adults were operationally defined as individuals aged 18–59 years, comprising young adults aged 18–44 years and middle-aged adults aged 45–59 years [14]. (ii) Acute stroke confirmed by imaging (computed tomography [CT] or magnetic resonance imaging [MRI]) [15,16], including ischemic or hemorrhagic stroke [17]. (iii) First-ever stroke, or patients with a prior stroke but complete clinical and functional assessment data for the current episode. (iv) Completion of at least 4 of the following 5 functional assessments during hospitalization, with complete data covering Barthel Index (BI) [18] and Mini-Mental State Examination (MMSE), Fugl-Meyer Assessment (FMA), Berg Balance Scale (BBS), and Numeric Rating Scale (NRS) for pain. (v) Availability of continuous and traceable physician orders and nursing/rehabilitation records during hospitalization to allow assessment of HBA.

#### 2.2.2 Exclusion Criteria

(i) Persistent coma or prolonged vegetative state at admission, preventing functional assessment and lacking reliable caregiver information. (ii) Prior diagnosis of severe dementia or major psychiatric disorders that could compromise the accuracy of adherence assessment. (iii) Concurrent end-stage malignancy or terminal illness with an expected survival of less than 3 months. (iv) Severe missing key clinical data, functional assessment scores, or nursing records, making it impossible to evaluate HBA.

#### 2.2.3 Study Sample

Based on the above criteria, 327 young and middle-aged stroke patients hospitalized between January 2021 and June 2024 were initially screened. Exclusions were: 19 patients with incomplete records, 8 patients unable to complete baseline cognitive assessment, and 2 patients with severe psychiatric disorders, totaling 29 exclusions. Finally, 298 patients were included in the statistical analysis.

### 2.3 Data Sources and Assessment Time Points

All data were obtained from the hospital electronic medical record system and routine functional assessment records. No additional questionnaires or assessments were administered, and no extra burden was placed on patients.

Baseline Assessment: Functional assessment results obtained within 48–72 hours after admission were considered baseline values, including BI, MMSE, FMA, BBS, and NRS.

Functional, cognitive, motor, balance, and pain assessments recorded at admission were used in the analyses. Stroke subtype was determined according to imaging-confirmed diagnosis documented in the electronic medical record and categorized as ischemic or hemorrhagic stroke.

### 2.4 Scales and Definition of Key Variables

#### Functional and Cognitive Assessment Scales

BI evaluates independence in 10 activities of daily living (ADL, e.g., feeding, bathing, dressing, toileting, transfers, ambulation). Scores range from 0 to 100, with higher scores indicating greater independence. BI was analyzed as a continuous variable, with standard reference categories: ≤40 points = severe dependence, 41–60 points = moderate dependence, 61–99 points = mild dependence, 100 points = complete independence [18].

MMSE assesses overall cognitive function, including orientation, memory, attention and calculation, language, and visuoconstruction, with a total score of 0–30. Lower scores indicate cognitive impairment [19]. MMSE total score was analyzed as a continuous variable.

FMA comprehensively evaluates upper- and lower-extremity motor function. The motor domain has a total score ranging from 0 to 100, comprising 66 points for the upper extremity and 34 points for the lower extremity. Higher scores indicate better motor function. The total FMA motor score was analyzed as a continuous variable [20].

BBS consists of 14 items assessing balance (e.g., sit-to-stand, single-leg stance, turning, forward reach), scored 0–56. Higher scores indicate better balance [21].

NRS: Self-reported pain scale ranging from 0 (no pain) to 10 (unbearable pain). Baseline NRS was recorded from early hospitalization [22,23].

### 2.5 HBA Outcome

#### 2.5.1 Operational Definition of HBA

HBA during hospitalization was defined as patients’ execution of physician orders and health-related guidance, including: (1) Medication adherence: Timely and correct intake of secondary prevention medications (antiplatelet/anticoagulants, antihypertensives, lipid-lowering, antidiabetic agents), without refusal, missed doses, or self-discontinuation. (2) Rehabilitation training adherence: Compliance with position management, active/passive limb exercises, sitting up, getting out of bed, and walking as prescribed by medical and rehabilitation staff. (3) Risk prevention adherence: Execution of measures to prevent falls, pressure ulcers, and deep vein thrombosis (e.g., use of assistive devices, adherence to “call for assistance before getting out of bed” rules).

#### 2.5.2 Quantification and Grouping of Adherence

To enhance the reliability and reproducibility of adherence assessment, the HBA Index was developed specifically for this study based on routinely documented clinical information during hospitalization. Because no universally validated instrument or weighting system currently exists for evaluating inpatient health behavior adherence after stroke, we constructed a study-specific exploratory index incorporating three clinically relevant domains: medication adherence, rehabilitation participation, and risk-prevention behavior adherence.

Adherence scoring was performed independently by two trained researchers using a standardized scoring manual developed a priori. The manual provided explicit operational definitions and representative examples for each scoring level to minimize subjective interpretation during medical record abstraction. Each documented adherence assessment within the three domains was scored on a three-level scale (2 = full adherence or active cooperation; 1 = partial adherence or occasional noncompliance; 0 = explicit refusal or repeated noncompliance). The scores obtained from the available assessments during hospitalization were aggregated across the three domains and standardized as a percentage.

Each domain was assigned equal weighting because there is currently insufficient evidence to determine that any single behavioral domain is superior to others in hospitalized stroke patients. This approach was adopted to reduce subjective weighting bias and improve reproducibility. The HBA Index was calculated as follows:

HBA Index (%) = sum of scores obtained across all assessed adherence items / maximum possible total score for the corresponding assessed items × 100%. 

Because the HBA Index was calculated from multiple documented assessments rather than from a single 0–2 score for each domain, it could take a range of continuous percentage values. Based on the calculated HBA Index, patients were categorized into a high-adherence group (≥80%) and a non-high-adherence group (<80%). In subsequent logistic regression analyses and predictive modeling, non-high adherence was defined as the primary outcome (0 = high adherence, 1 = non-high adherence).

Before formal data extraction, both raters completed calibration training and pilot scoring using a subset of medical records to ensure consistent application of the scoring criteria. During the full assessment process, all records were independently reviewed by both investigators. Any disagreement was resolved through discussion, with adjudication by a senior investigator when necessary.

To evaluate the reproducibility of the HBA Index, inter-rater reliability analysis was performed. The intraclass correlation coefficient (ICC) was calculated for continuous HBA scores, while Cohen’s kappa coefficient was used to assess agreement in adherence classification. The continuous HBA scores demonstrated excellent inter-rater reliability (ICC = 0.972), and the agreement for adherence classification was also excellent (Cohen’s κ = 0.933; overall agreement rate = 96.6%).

### 2.6 Statistical Analysis

All analyses were performed using statistical software (SPSS: SPSS version 26.0 (IBM Corp., Armonk, NY, USA) or R: R software version 4.3.2 (R Foundation for Statistical Computing, Vienna, Austria)). Continuous variables are expressed as mean ± standard deviation or median (interquartile range), and categorical variables as counts and percentages. Patients were first divided into high- and non-high-adherence groups. Group differences in demographics, clinical features, and functional scores were analyzed using independent-samples *t*-test for normally distributed continuous variables, Mann–Whitney U test for non-normal variables, and χ^2^ test or Fisher’s exact test for categorical variables. Pearson or Spearman correlation analyses were conducted (depending on data distribution) to assess associations between adherence index and BI, MMSE, FMA, BBS, and NRS. Candidate variables for multivariable logistic regression analysis were selected based on both statistical significance in univariate analysis (*p* < 0.10) and clinical relevance. Considering that several functional assessments may reflect overlapping aspects of post-stroke recovery, collinearity diagnostics were performed before model construction. Variables representing highly correlated clinical domains were carefully evaluated, and representative variables were retained to improve model stability and interpretability. Multicollinearity among candidate predictors was assessed using variance inflation factors (VIFs), with VIF values <10 considered acceptable. BI, MMSE, FMA, and BBS exhibited substantial multicollinearity when simultaneously entered into the initial model. To avoid coefficient instability and redundant representation of closely related functional measures, MMSE was retained on the basis of its clinical relevance to adherence behavior and its role in the proposed predictive model, whereas BI, FMA, and BBS were excluded from the final multivariable model. Multicollinearity was reassessed after model specification. All predictors retained in the final model had VIF values below (maximum VIF 6.94). Model calibration was evaluated using the Hosmer–Lemeshow goodness-of-fit test, and influential observations were examined using standardized residuals, leverage values, and Cook’s distance. For variables representing similar clinical domains, representative variables were selected to avoid redundancy and potential multicollinearity. Odds ratios (ORs) and 95% confidence intervals (95% CIs) were calculated. Significant variables from multivariate logistic regression were used to construct a combined predictive model. Receiver operating characteristic (ROC) curves were plotted, and area under the curve (AUC), Youden index, sensitivity, and specificity were calculated to evaluate predictive performance of individual indicators and the combined model. For the combined predictive model, the optimal cutoff value was determined according to the maximum Youden index. Classification performance was further evaluated by calculating sensitivity, specificity, positive predictive value, negative predictive value, and diagnostic accuracy. All tests were two-tailed, with *p* < 0.05 considered statistically significant. Internal validation of the predictive model was performed using bootstrap resampling with 1000 repetitions. The optimism-corrected AUC was calculated by estimating the average difference between bootstrap model performance and performance in the original sample. Model calibration was evaluated by comparing predicted probabilities with observed outcomes using calibration plots. Predicted probabilities were grouped into deciles to assess the agreement between expected and observed risks. Model calibration was assessed using calibration plots and the Hosmer–Lemeshow goodness-of-fit test. A non-significant Hosmer–Lemeshow test result (*p* > 0.05) was considered indicative of acceptable model calibration. Missing data patterns were examined before statistical analysis. Pearson’s correlation analysis was used when the assumptions of approximate normality and linearity were satisfied; otherwise, Spearman’s rank correlation analysis was used. Because no missing values were observed among the variables included in the final analyses, complete-case analysis was applied and no missing data imputation was performed.

### 2.7 Sample Size and Model Complexity

The primary outcome was non-high adherence (1 = non-high adherence, 0 = high adherence). Multivariate logistic regression requires at least 10–15 events per variable (EPV ≥10) to reduce overfitting and ensure stable parameter estimates.

This study included approximately 6 candidate variables (BI, MMSE, FMA, BBS, NRS, and educational level), requiring a minimum of 60–90 non-high-adherence events and a total sample size of 160–240 patients. The actual sample included 298 patients, with 143 (48.0%) non-high-adherence cases, exceeding EPV requirements and supporting at least 8 variables in the multivariate model.

Based on classical logistic regression power analysis, with a 50% event rate, a sample size ~300, and α = 0.05 (two-sided), continuous variables with OR ≈ 1.6–1.8 (per 1 standard deviation change) achieve ~80% power, and OR ≈ 2.0 achieves ~90% power. Therefore, this study could reliably detect moderate or higher effect sizes (e.g., NRS, educational level OR 1.6–2.1), with stable effect estimates for core variables such as functional and cognitive scores.

Candidate predictors were initially entered into a multivariable logistic regression model. Because BI, MMSE, FMA, and BBS exhibited substantial multicollinearity, BI, FMA, and BBS were excluded, while MMSE was retained on the basis of its clinical relevance to adherence behavior. The model was then refitted using MMSE, NRS, educational level, and first-stroke status. This four-predictor model was designated as the final parsimonious predictive model.

ROC analysis is also supported. With 143 events and 155 non-events, using the Hanley–McNeil formula at α = 0.05, power to distinguish AUC = 0.70–0.75 from no discrimination (AUC = 0.5) is ~0.85–0.90. The final predictive model demonstrated an apparent AUC of 0.850, and internal bootstrap validation showed an optimism-corrected AUC of 0.843, supporting acceptable discrimination performance. Therefore, the sample size is adequate for multivariate modeling and predictive performance assessment.

## 3. Results

### 3.1 Patient Inclusion and Grouping

A total of 327 young and middle-aged stroke patients were initially screened. According to the inclusion and exclusion criteria, 19 patients with incomplete records, 8 patients unable to complete baseline cognitive assessment, and 2 patients with severe psychiatric disorders were excluded, leaving 298 patients for analysis. Based on the in-hospital HBA Index, 155 patients were classified as high adherence and 143 as non-high adherence. The patient selection flow is shown in Fig. 1. After exclusion of patients with incomplete essential clinical information, 298 patients were included in the final analysis. All variables included in the predictive model were completely recorded, and no missing data were present among the analyzed variables.

**Fig. 1.**
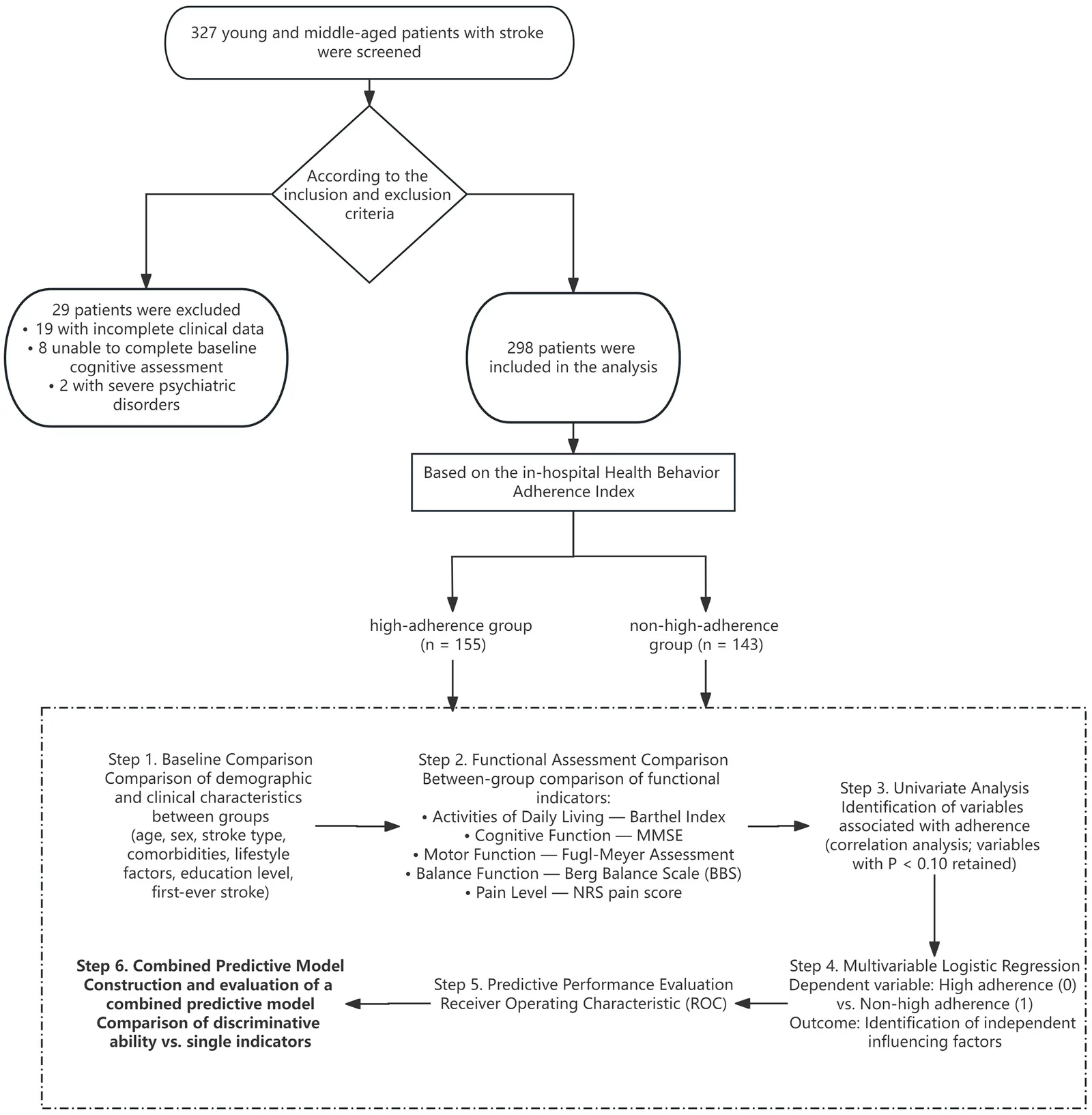
**Flowchart of patient selection and analysis**. A total of 327 patients were screened, and 298 were finally included. Patients were divided into high-adherence and non-high-adherence groups based on the in-hospital HBA Index. Baseline comparison, functional comparison, univariate analysis, multivariate logistic regression, and predictive model evaluation were performed sequentially. HBA, health behavior adherence; MMSE, Mini-Mental State Examination; FMA, Fugl-Meyer Assessment; NRS, Numeric Rating Scale.

### 3.2 Baseline Demographics and Clinical Characteristics

Among the 298 patients, 155 (52.0%) were in the high-adherence group and 143 (48.0%) in the non-high-adherence group. No significant differences were observed between the groups in age, sex distribution, stroke type, or history of hypertension (all *p* > 0.05), indicating good comparability of baseline clinical characteristics. However, educational level and first-ever stroke status differed significantly between groups. The proportion of patients with senior high school or above education was higher in the high-adherence group (59.35% vs. 39.86%, χ^2^ = 11.307, *p* = 0.001), and the proportion of first-ever stroke patients was also higher in the high-adherence group (76.13% vs. 62.24%, χ^2^ = 6.766, *p* = 0.009, Table 1). These results suggest that educational level and first-ever stroke may be potential factors influencing HBA.

**Table 1.** **Comparison of general demographics and clinical characteristics between the two groups**.

Characteristics	High-adherence group (n = 155)	Non-high-adherence group (n = 143)	*Z/χ^2^*	*p*
Age (years)	49 (45, 53)	50 (45, 53)	–0.949	0.343
Male (case, %)	104 (67.1%)	92 (64.34%)	0.252	0.616
Senior high school diploma or above (%)	92 (59.35%)	57 (39.86%)	11.307	0.001
First-ever stroke (%)	118 (76.13%)	89 (62.24%)	6.766	0.009
Stroke subtype-Ischemic stroke (%)	121 (78.06%)	108 (75.52%)	0.270	0.604
Stroke subtype-Hemorrhagic stroke	34 (21.94%)	35 (24.48%)		
Hypertension history (%)	89 (57.42%)	87 (60.84%)	0.360	0.549

Note: Categorical variables are presented as n (%). Non-normally distributed data were analyzed using rank-sum test; categorical variables were analyzed using χ^2^ test. *p* < 0.05 indicates statistical significance.

### 3.3 Comparison of Functional Scores Between Groups

Regarding functional and symptom indicators, patients in the high-adherence group demonstrated significantly better activity, cognitive, motor, and balance function, and lower pain levels compared with the non-high-adherence group (Table 2). Specifically, BI was higher in the high-adherence group [83 (73, 91) vs. 75 (63, 82), Z = –6.557, *p* < 0.001]. MMSE scores were higher in the high-adherence group [27 (25, 29) vs. 23 (21, 26), Z = –8.797, *p* < 0.001]. FMA scores were higher in the high-adherence group (76.07 ± 12.93 vs. 66.78 ± 15.39, t = –5.621, *p* < 0.001). BBS scores were higher in the high-adherence group [46 (40, 51) vs. 39 (33, 45), Z = –6.198, *p* < 0.001]. NRS scores were lower in the high-adherence group [2 (2, 3) vs. 3 (2, 4), Z = 4.937, *p* < 0.001]. These results indicate that better functional status, cognitive ability, balance function, and lower pain burden are associated with higher in-hospital HBA.

**Table 2.** **Comparison of functional and symptom indicators between the two groups**.

Indicators	High-adherence group (n = 155)	Non-high-adherence group (n = 143)	*t/Z*	*p*
Barthel Index (score)	83 (73, 91)	75 (63, 82)	–6.557	<0.001
MMSE (score)	27 (25, 29)	23 (21, 26)	–8.797	<0.001
FMA (score)	76.07 ± 12.93	66.78 ± 15.39	–5.621	<0.001
BBS (score)	46 (40, 51)	39 (33, 45)	–6.198	<0.001
NRS score	2 (2, 3)	3 (2, 4)	4.937	<0.001

Note: Normally distributed data are presented as mean ± SD and analyzed using *t*-test; non-normally distributed data are presented as median (interquartile range) and analyzed using rank-sum test. *p* < 0.05 indicates statistical significance. SD, Standard Deviation; BBS, Berg Balance Scale.

### 3.4 Correlation Between Adherence and Functional/Symptom Indicators

Correlation analysis revealed significant linear associations between HBA and multiple functional indicators (Fig. 2). Adherence was positively correlated with BI (Spearman’s r_s_ = 0.4975, *p* < 0.0001), MMSE (Spearman’s r_s_ = 0.5866, *p* < 0.0001), FMA (Pearson’s r = 0.4210, *p* < 0.0001), and BBS (Spearman’s r_s_ = 0.4489, *p* < 0.0001), and negatively correlated with NRS (Spearman’s r_s_ = –0.4032, *p* < 0.0001). These findings suggest that patients with better functional status, cognitive function, motor and balance ability, and lower pain levels exhibit higher in-hospital adherence.

**Fig. 2.**
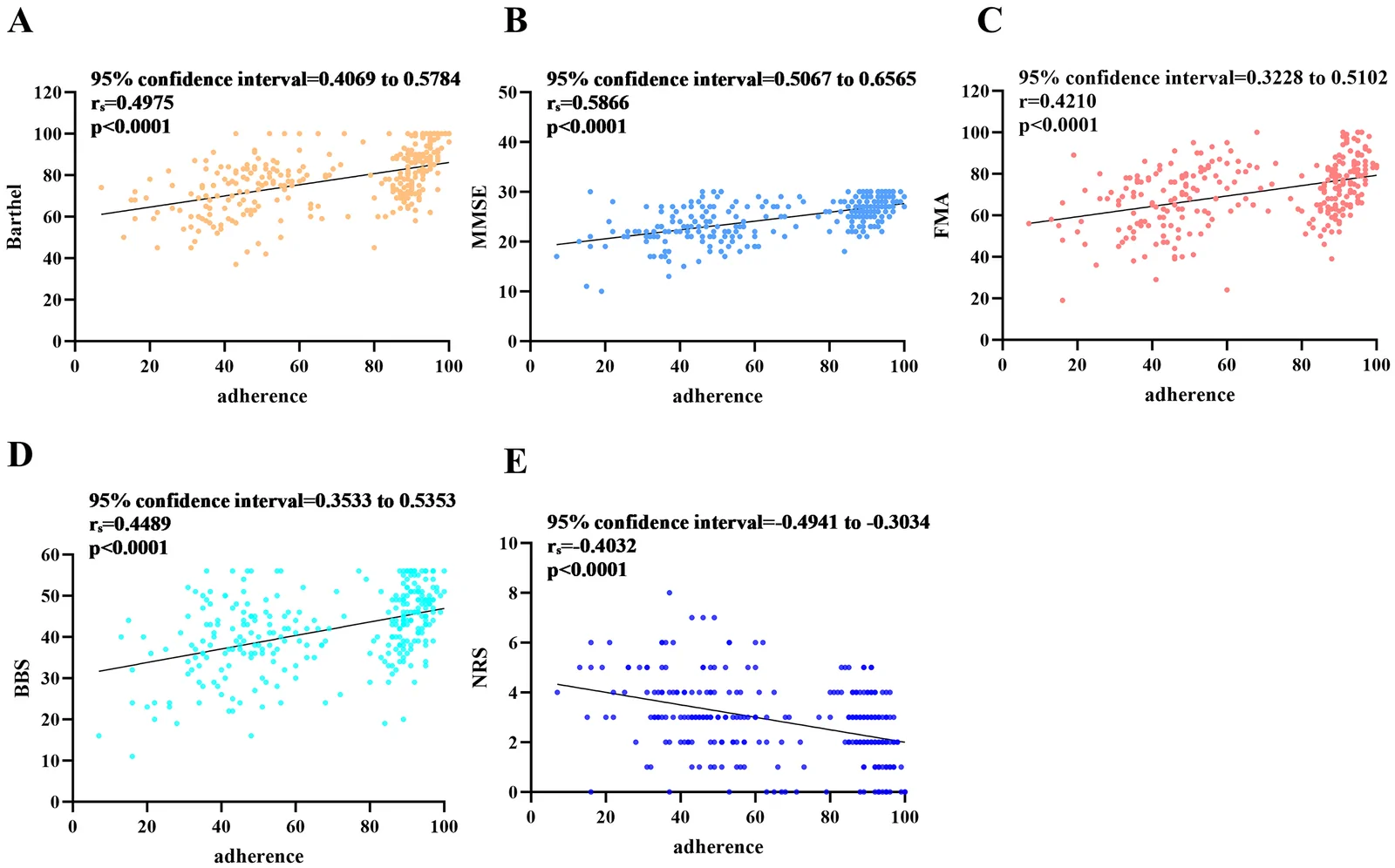
**Correlation analysis between adherence and functional/symptom indicators**. (A) Adherence vs. BI. (B) Adherence vs. MMSE. (C) Adherence vs. FMA. (D) Adherence vs. BBS. (E) Adherence vs. NRS, with 95% CI shown. BI, Barthel Index. The HBA Index represents the standardized percentage score derived from the summed item-level adherence assessments across the three domains during hospitalization.

### 3.5 Multivariate Logistic Regression Analysis

Using “non-high adherence” as the outcome variable (1 = non-high adherence, 0 = high adherence), variables with statistical significance in univariate analysis were entered into a multivariate logistic regression model. Results showed that poorer functional and cognitive scores were strongly associated with increased risk of non-high adherence (Tables 3,4). BI (OR = 0.921, 95% CI: 0.891–0.952, *p* < 0.001), MMSE (OR = 0.643, 95% CI: 0.564–0.732, *p* < 0.001), FMA (OR = 0.944, 95% CI: 0.919–0.969, *p* < 0.001), and BBS (OR = 0.902, 95% CI: 0.863–0.943, *p* < 0.001) were independent factors associated with lower likelihood of non-high adherence, with each 1-point increase reducing the risk of non-high adherence by approximately 7.9%, 35.7%, 5.6%, and 9.8%, respectively. Higher pain significantly increased the risk of non-high adherence (NRS: OR = 1.601, 95% CI: 1.255–2.042, *p* < 0.001). Lower educational level and recurrent-stroke status were independently associated with higher odds of non-high adherence (OR = 2.149, 95% CI: 1.042–4.433, *p* = 0.038). First-ever stroke was not significant in the multivariate model (*p* = 0.124). Overall, limited functional status, poorer cognitive ability, inadequate balance, higher pain, and lower educational level were independent predictors of non-high adherence among young and middle-aged stroke patients during hospitalization.

**Table 3.** **Variable coding for logistic regression**.

Variables	Value coding
BI	Continuous variables
MMSE	Continuous variables
FMA	Continuous variables
BBS	Continuous variables
NRS	Continuous variables
Educational level	Senior high school diploma or above = 0; Below senior high school diploma = 1
First-ever stroke	Yes = 0; No = 1

**Table 4.** **Multivariate logistic regression analysis of factors affecting HBA in young and middle-aged stroke patients**.

Variable	β	SE	Wald	*p*-value	OR [95% CI (lower limit, upper limit)]	Standardized OR (per SD increase)	VIF
BI	–0.082	0.017	24.202	<0.001	0.921 (0.891–0.952)	0.329	29.44
MMSE	–0.442	0.066	44.566	<0.001	0.643 (0.564–0.732)	0.187	35.62
FMA	–0.058	0.013	18.793	<0.001	0.944 (0.919–0.969)	0.422	21.20
BBS	–0.103	0.022	21.091	<0.001	0.902 (0.863–0.943)	0.389	21.20
NRS	0.47	0.124	14.357	<0.001	1.601 (1.255–2.042)	2.046	3.88
Educational level	0.765	0.369	4.289	0.038	2.149 (1.042–4.433)	-	2.11
First-ever stroke	0.625	0.407	2.361	0.124	1.868 (0.842–4.147)	-	3.45
Constant	23.844	3.152	57.236	<0.001	-	-	-

BI, MMSE, FMA, and BBS were independent factors associated with a lower likelihood of non-high adherence; NRS and lower educational level were independent associated factors; first-ever stroke was not significant in the model. OR, odds ratio; CI, confidence interval; SE, standard error; VIF, variance inflation factor.

### 3.6 Predictive Performance of Single Indicators and Combined Model

ROC analysis demonstrated that multiple functional and symptom indicators could discriminate non-high adherence (Table 5, Fig. 3). Among single indicators, MMSE had the best discriminatory performance (AUC = 0.7938, 95% CI: 0.7428–0.8447; Youden index = 0.4767; sensitivity = 65.73%; specificity = 81.94%). BI and BBS had moderate performance (AUC = 0.7197 [95% CI: 0.6624–0.7770] and 0.7076 [95% CI: 0.6487–0.7665]; Youden index = 0.3521 and 0.3347). FMA and NRS had relatively limited performance (AUC = 0.6681 and 0.6621). Educational level and first-ever stroke had low AUCs (0.5975 and 0.5695), indicating weak predictive ability alone. The model, incorporating significant variables from multivariate logistic regression, achieved an AUC of 0.9291 (95% CI: 0.9006–0.9575; Youden index = 0.7661), substantially higher than any single indicator. Sensitivity and specificity were both high, demonstrating excellent discriminatory performance and supporting its clinical utility for identifying patients at high risk of non-high adherence. At the optimal probability cutoff of 0.475, determined by maximizing the Youden index, the final model achieved a sensitivity of 89.51%, a specificity of 87.10%, a positive predictive value of 86.49%, a negative predictive value of 90.00%, and an overall accuracy of 88.26%. These findings indicate that the combined model showed favorable classification performance for identifying patients with non-high adherence. To evaluate the robustness of the predictive model, internal validation was performed using bootstrap resampling with 1000 repetitions. The original model showed an AUC of 0.9291, and the estimated optimism was 0.0077. After optimism correction, the combined model maintained a stable discrimination performance with a corrected AUC of 0.9214, indicating limited optimism and good model stability. In addition to discrimination performance, model calibration was assessed.

**Table 5.** **Predictive performance of single indicators and combined model for non-high adherence in young and middle-aged stroke patients**.

Variable	AUC	Youden	SE	95% CI	Sensitivity (%)	Specificity (%)
BI	0.7197	0.3521	0.02924	0.6624 to 0.7770	88.11	47.10
MMSE	0.7938	0.4767	0.02602	0.7428 to 0.8447	65.73	81.94
FMA	0.6681	0.2438	0.03099	0.6074 to 0.7289	56.64	67.74
BBS	0.7076	0.3347	0.03006	0.6487 to 0.7665	65.73	67.74
NRS	0.6621	0.2606	0.03159	0.6002 to 0.7240	69.93	56.13
Educational level	0.5975	0.1949	0.03288	0.5330 to 0.6619	60.14	59.35
First stroke	0.5695	0.1389	0.03328	0.5042 to 0.6347	37.76	76.13
Model	0.9291	0.7661	0.01451	0.9006 to 0.9575	89.51	87.10

Note: AUC, Youden index, SE, 95% CI, sensitivity, and specificity are shown for BI, MMSE, FMA, BBS, NRS, education level, first-ever stroke, and model. AUC, Area Under Curve.

**Fig. 3.**
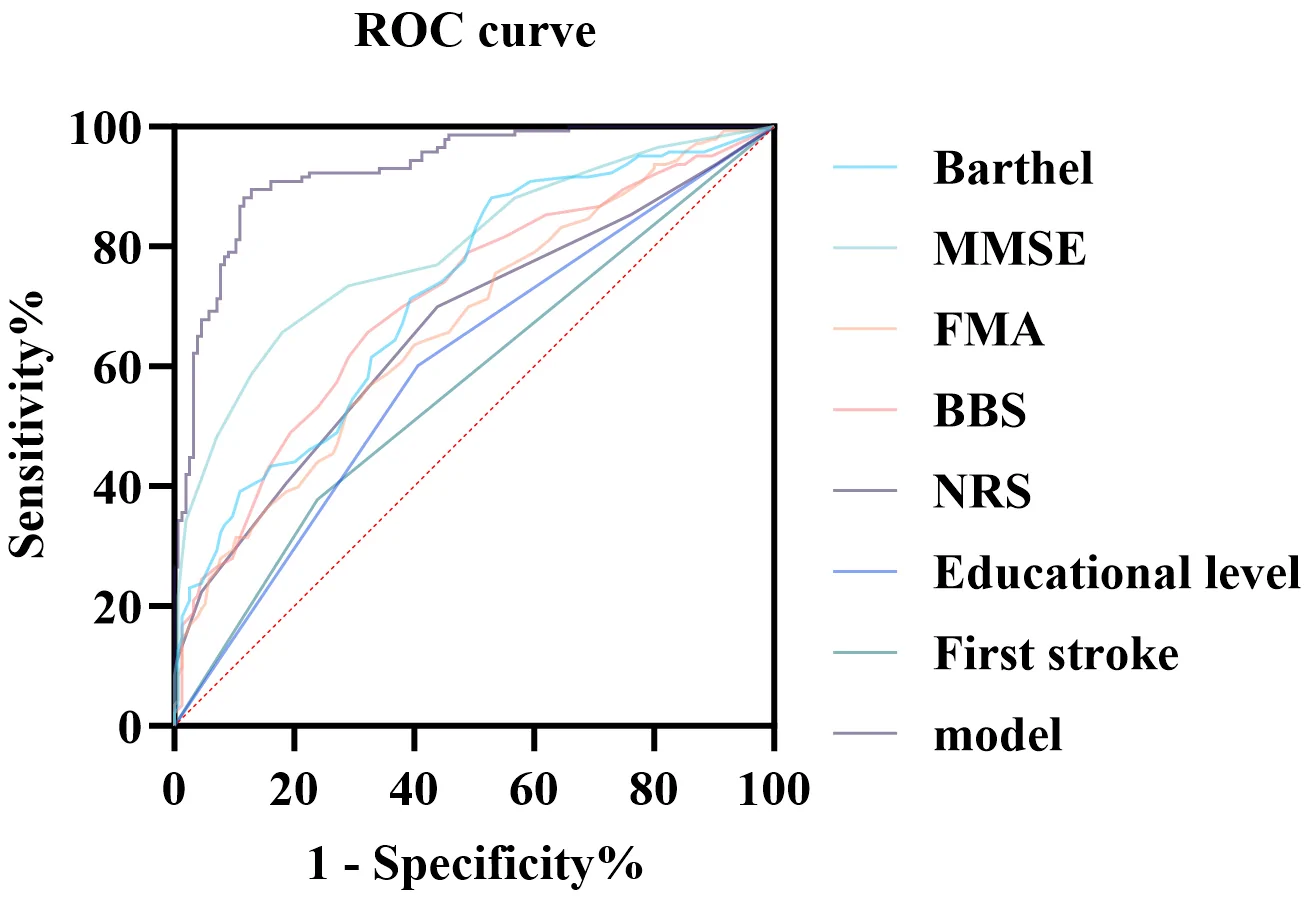
**ROC curves of single indicators and combined model for non-high adherence**. The curves for BI, MMSE, FMA, BBS, NRS, education level, first-ever stroke, and the combined predictive model are shown. Receiver operating characteristic curves for the individual predictors and the seven-predictor combined model. The combined model included BI, MMSE, FMA, BBS, NRS, educational level, and first-stroke status.

The calibration plot demonstrated good agreement between predicted and observed probabilities of non-high adherence, indicating satisfactory calibration of the combined predictive model (Fig. 4). In addition to discrimination performance, model calibration was evaluated. The Hosmer–Lemeshow goodness-of-fit test showed no significant deviation between predicted and observed outcomes (χ^2^ = 12.043, df = 8, *p* = 0.149), indicating satisfactory calibration of the combined model. Assessment of influential observations did not identify any highly influential cases affecting model estimates.

**Fig. 4.**
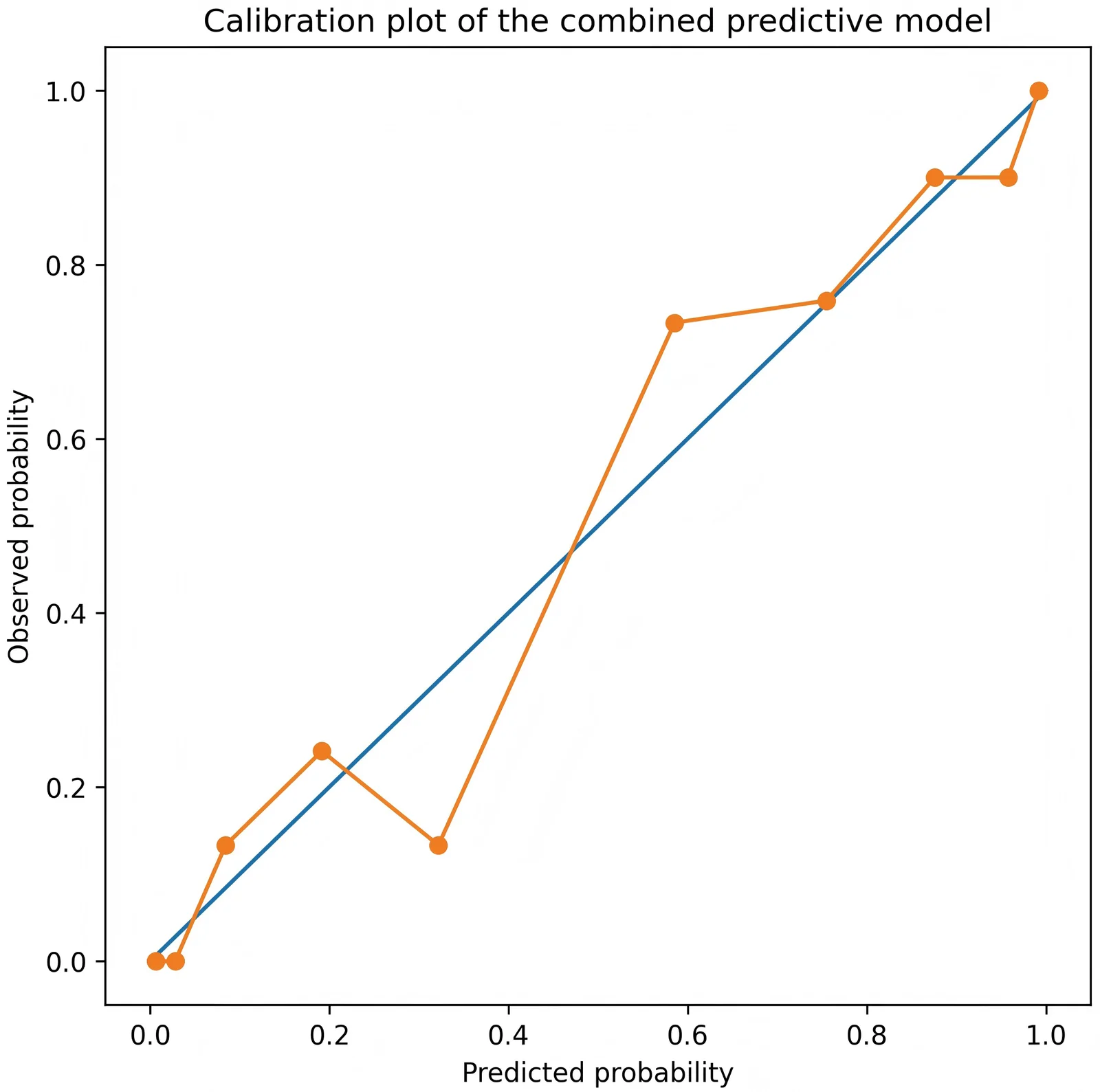
**Calibration plot of the combined predictive model for predicting non-high adherence in young and middle-aged stroke patients**. The calibration plot compares predicted probabilities with observed outcomes across deciles of predicted risk. The diagonal line represents perfect calibration, whereas the calibration curve demonstrates the agreement between predicted and observed probabilities. The model showed satisfactory calibration performance.

### 3.7 Sensitivity Analysis of the Predictive Model

To minimize potential multicollinearity among overlapping clinical variables, a simplified multivariable logistic regression model incorporating MMSE, NRS, education level, and first stroke status was established. Higher MMSE scores were independently associated with a lower likelihood of non-high adherence (OR = 0.685, 95% CI: 0.619–0.758, *p* < 0.001), whereas higher pain intensity was associated with increased odds of non-high adherence (OR = 1.651, 95% CI: 1.349–2.022, *p* < 0.001, Table 6). Higher educational level (OR = 0.490, 95% CI: 0.273–0.882, *p* = 0.017) and first-ever stroke (OR = 0.404, 95% CI: 0.214–0.763, *p* = 0.005) were associated with reduced odds of non-high adherence. Calibration analysis showed good agreement between predicted and observed probabilities, with a calibration intercept of 0.003 and slope of 1.020 (Fig. 5A). The Hosmer–Lemeshow test demonstrated satisfactory model calibration (χ^2^ = 9.546, df = 8, *p* = 0.298). The initial model, which included correlated functional and cognitive measures, yielded an AUC of approximately 0.929 but was not retained because of substantial multicollinearity. After removal of the redundant predictors, the final parsimonious model included MMSE, NRS, educational level, and first-stroke status and yielded an AUC of 0.850, indicating good discrimination. All subsequent references to the combined or final model refer to this four-predictor model (Fig. 5B). Based on the optimal cutoff value determined by the maximum Youden index, the model achieved a sensitivity of 80.42%, specificity of 76.77%, positive predictive value (PPV) of 76.16%, negative predictive value (NPV) of 80.95%, and accuracy of 78.52%. After bootstrap internal validation with 1000 repetitions, the optimism-corrected AUC remained 0.843, indicating stable predictive performance.

**Table 6.** **Multivariable logistic regression analysis of factors associated with non-high adherence in young and middle-aged stroke patients**.

Variable	B	SE	Wald	OR (95% CI)	*p* value	Standardized OR (per SD increase)	VIF
MMSE	−0.379	0.052	53.98	0.685 (0.619–0.758)	<0.001	0.237	6.94
NRS	0.502	0.103	23.56	1.651 (1.349–2.022)	<0.001	2.149	3.81
Education level	−0.713	0.3	5.68	0.490 (0.273–0.882)	0.017	-	2.11
First stroke	−0.906	0.325	7.79	0.404 (0.214–0.763)	0.005	-	3.30

The dependent outcome was coded as 1 = non-high adherence and 0 = high adherence. Educational level was coded as 1 = below high school and 0 = high school or above. First-stroke status was coded as 0 = first-ever stroke and 1 = recurrent stroke. Therefore, positive regression coefficients indicate higher odds of non-high adherence for patients with education below high school and for patients with recurrent stroke, respectively.

**Fig. 5.**
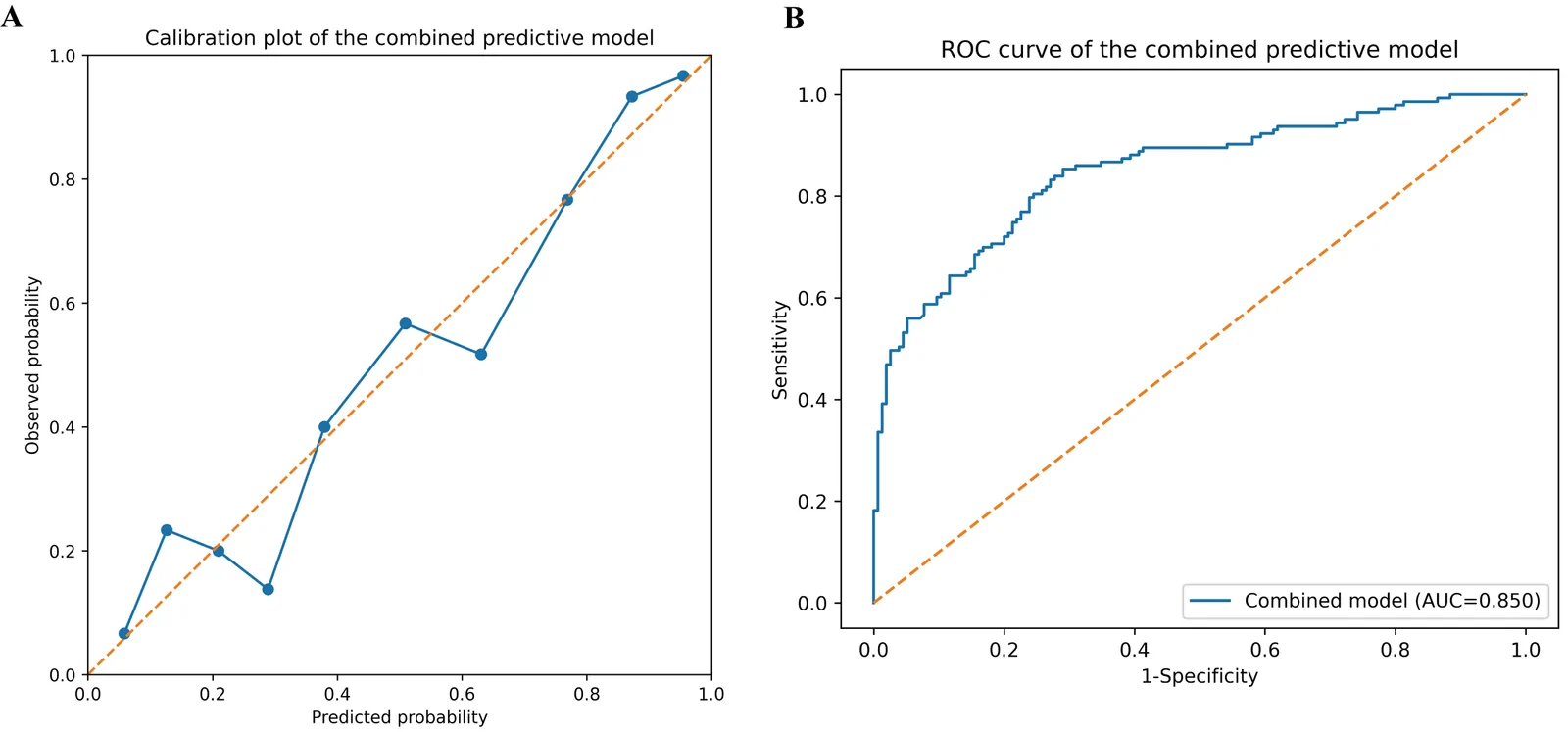
**Calibration plot of the combined predictive model for predicting non-high adherence in young and middle-aged stroke patients (final model)**. (A) The calibration plot compares predicted probabilities with observed outcomes across deciles of predicted risk. The diagonal line represents perfect calibration, whereas the calibration curve demonstrates the agreement between predicted and observed probabilities. The model showed satisfactory calibration performance. (B) ROC curve of the combined predictive model for non-high adherence in young and middle-aged stroke patients.

## 4. Discussion

This study systematically evaluated in-hospital HBA and its influencing factors in young and middle-aged stroke patients. Results showed that nearly half of patients exhibited non-high adherence, indicating that suboptimal adherence is common in this population. Further analysis revealed that functional status, cognitive ability, balance, pain intensity, and education level are key factors affecting adherence [24]. Specifically, BI, FMA, BBS, and MMSE were important factors associated with lower likelihood of non-high adherence against non-high adherence, whereas NRS and lower education level were independent associated factors. Correlation analysis and multivariate logistic regression were highly consistent, and the combined predictive model yielded an AUC, significantly outperforming any single indicator, suggesting that integrating multidimensional information including function, cognition, and pain can markedly enhance risk identification. These findings provide a clear clinical and behavioral evidence chain regarding the mechanisms underlying adherence in young and middle-aged stroke patients and offer an important basis for targeted interventions. Cognitive function may represent an important determinant associated with health behavior adherence after stroke. Although cognitive impairment may influence information processing, decision-making, and self-management behaviors, the causal pathways underlying this association remain to be elucidated. The predictive model should therefore be interpreted as a clinically available model based on routinely collected variables rather than a comprehensive behavioral model incorporating all potential determinants of adherence.

### 4.1 Comparison With Previous Studies

Most previous studies focused on older stroke populations, reporting that cognitive impairment, functional limitation, and insufficient social support were associated with decreased adherence [25], while systematic studies in young and middle-aged patients remain limited. Our findings are partly consistent with prior evidence, indicating that better function and greater capacity for task execution and active participation are associated with higher adherence [26]. MMSE demonstrated the highest discriminative ability among single indicators in this study. One possible explanation is that impaired cognitive function may affect patients’ understanding of health information, decision-making capacity, and engagement in self-management behaviors. However, this hypothesis requires further validation in prospective studies incorporating detailed neuropsychological and behavioral assessments. It influences comprehension of medical orders, risk perception, and behavioral planning, thereby affecting adherence levels. This finding supports the “cognition–behavioral execution–adherence” pathway hypothesis in behavioral medicine, providing a mechanistic explanation for adherence differences.

Although pain is a common post-stroke complication, it has not been specifically evaluated as a determinant in several major reviews of medication adherence after stroke [27,28,29]. This suggests that pain may continuously suppress adherence via a “physiological–psychological–behavioral” pathway by limiting activity tolerance, reducing rehabilitation engagement, and inducing negative emotional responses. Education level was also significant, as patients with lower education are more likely to have limited health knowledge, low risk awareness, and reduced health literacy, making sustained adherence challenging. These results suggest that adherence formation in young and middle-aged stroke patients depends not only on physical recovery but also on cognitive capacity, emotional experience, and access to social resources, reflecting a multidimensional and interactive mechanism.

### 4.2 Model Innovations

Compared with previous studies, this study has several innovations. First, it focuses on the “young and middle-aged” population, which is often overlooked but clinically important, addressing the limitation of adherence research biased toward older populations. Second, adherence assessment was not solely based on self-reported questionnaires but constructed as an objective HBA index using medical order execution, nursing records, and rehabilitation documentation, enhancing assessment validity and traceability. Third, by integrating function, cognition, pain, and education into a combined predictive model, this study not only identified key influencing factors but also achieved high accuracy in risk prediction, significantly outperforming traditional univariate approaches. This methodological framework is clinically replicable and scalable, providing a novel pathway for adherence analysis and prediction. Several potential mechanisms may explain the observed association between cognitive function and HBA. Cognitive impairment may reduce patients’ ability to understand health-related information, evaluate consequences, and actively participate in rehabilitation and secondary prevention behaviors. However, these explanations remain hypothetical because the present study was retrospective and observational. Future prospective studies integrating neuropsychological assessments, behavioral measurements, and longitudinal follow-up are needed to clarify whether cognitive function directly contributes to changes in health behavior adherence.

### 4.3 Clinical Significance and Translational Value

The findings have important clinical and nursing implications. First, HBA should be considered a core issue in stroke management, with early attention especially in young and middle-aged patients. Second, functional and cognitive assessments should be used not only as outcome indicators but also as screening tools for adherence risk, recommending early standardized evaluation and integration into adherence management pathways. Third, pain management and health education should be strengthened [7], with targeted and interactive guidance for patients with lower education levels to improve behavioral feasibility. Ultimately, the combined predictive model may facilitate early identification of patients at higher likelihood of non-high adherence and provide a potential basis for targeted intervention strategies, which can enhance rehabilitation engagement, improve functional recovery, and reduce recurrence risk, demonstrating significant translational potential.

### 4.4 Limitations and Future Directions

This study has several limitations. First, as a single-center retrospective observational study, selection bias and information bias cannot be completely excluded. Although bootstrap internal validation demonstrated stable discrimination performance, the predictive model was developed and internally validated within a single-center dataset. Therefore, external validation in independent multicenter cohorts is required to confirm its generalizability and clinical applicability. Furthermore, because clinical characteristics and HBA assessments were obtained during the same hospitalization period, temporal relationships and causal associations could not be established. The observed associations should therefore be interpreted as hypothesis-generating rather than causal.

Second, although the HBA Index was developed using predefined criteria and multiple sources of clinical documentation, including medical orders, nursing records, rehabilitation records, and health education records, potential measurement bias remains possible. Variations in documentation completeness and clinical practice patterns may have influenced adherence classification. Future prospective studies incorporating standardized behavioral assessments, wearable devices, and digital health technologies may improve the objectivity and reproducibility of HBA measurement.

Third, although the predictive model incorporated several clinically relevant functional, cognitive, and demographic variables, residual confounding cannot be completely excluded. Important determinants of health behavior adherence, particularly psychosocial factors such as depression, anxiety, self-efficacy, health literacy, family support, and socioeconomic conditions, were not systematically available in this retrospective dataset. These factors may contribute substantially to adherence behaviors among young and middle-aged stroke patients. Future studies integrating multidimensional clinical, psychosocial, and behavioral assessments are needed to develop more comprehensive adherence prediction models.

Fourth, detailed stroke etiological subtype information based on the Trial of Org 10172 in Acute Stroke Treatment (TOAST) classification was unavailable for all patients. Therefore, potential differences in HBA according to specific stroke mechanisms, including cardioembolism, large artery atherosclerosis, and small vessel occlusion, could not be evaluated. Because lacunar and non-lacunar strokes differ in pathophysiology, clinical characteristics, and prognosis, future multicenter studies incorporating standardized stroke subtype classification should investigate whether determinants of HBA vary across different stroke mechanisms [30].

Fifth, the HBA Index was developed as a study-specific exploratory measure because no universally accepted weighting system currently exists for inpatient stroke-related adherence assessment. Although equal weighting was applied to minimize subjective bias and improve reproducibility, the relative contribution of different behavioral domains may vary among individuals. Future studies should further validate the HBA Index and explore data-driven weighting strategies to optimize its predictive performance.

Finally, because functional indicators such as BI, FMA, and BBS reflect overlapping aspects of post-stroke recovery, the model specification was refined to reduce redundancy and improve interpretability. However, larger multicenter studies with comprehensive variable collection and advanced modeling approaches are needed to further optimize predictive performance. Cognitive function should be considered as one important factor associated with HBA, while adherence behavior is likely influenced by multiple interacting biological, psychological, and social determinants.

## 5. Conclusions

This study systematically evaluated in-hospital HBA and its influencing factors in young and middle-aged stroke patients, showing that nearly half of patients exhibited non-high adherence, highlighting its clinical significance. Functional status (BI, FMA, BBS), cognitive ability (MMSE), and pain intensity (NRS) were closely associated with adherence, with good functional and cognitive levels serving as factors associated with lower likelihood of non-high adherence, and pain and lower education as independent associated factors. ROC analysis further confirmed that a multivariate model integrating function, cognition, and education had excellent discriminative ability, clearly superior to any single indicator, effectively identifying high-risk patients for non-high adherence.

The findings indicate that in managing young and middle-aged stroke patients, adherence is not only a critical determinant of rehabilitation outcomes but also a modifiable clinical target. Enhancing functional rehabilitation guidance, prioritizing cognitive assessment and intervention, optimizing pain management, and providing targeted health education may significantly improve adherence behaviors and rehabilitation outcomes. This study provides scientific evidence for establishing an objective, risk-based adherence management system, contributing to precision stroke rehabilitation. Multicenter prospective studies are still needed to validate these findings and to develop more intelligent and individualized adherence intervention strategies.

## Data Availability

The data supporting the findings of this study are available from the corresponding author upon reasonable request.
